# Do long-term care facilities feel like home? Views from older adults with disabilities

**DOI:** 10.1186/s12877-026-07379-w

**Published:** 2026-03-25

**Authors:** Yuran Li, Ruping Zou, Junjie Niu, Mengshi Liu, Min Wu, Jingshuo Zhang, Xiaoman Zhang

**Affiliations:** School of Nursing, Xuzhou Medical University, No. 209 Tongshan Road, Xuzhou, Jiangsu Province 221004 China

**Keywords:** Home, Nursing home, Disabilities, Older adults, Long-term care, Care facilities

## Abstract

**Background:**

The sense of home for older adults with disabilities in long-term care facilities is a multifaceted phenomenon and a complex concept that can be challenging to grasp. This sense of home is crucial for helping this population maintain autonomy, security, and well-being. As older adults with disabilities often find themselves spending extended periods in long-term care due to physical limitations, understanding their perception of home in such settings can aid in creating appropriate, home-like environments in which they can experience higher levels of satisfaction, comfort and a sense of belonging.

**Aim:**

The purpose of this study was to explore and describe the lived experience of a sense of home among older adults with disabilities residing in long-term care facilities, and to understand the factors that shape this experience.

**Method:**

We employed the descriptive phenomenology method to purposefully select 16 older adults with disabilities from a long-term care facility in East China. Data was collected through semi-structured interviews and subsequently analyzed using Colaizzi’s analysis method.

**Findings:**

The analysis of the study identified five themes: (a)self-identity, (b)gaining a sense of belonging, (c) a warm atmosphere, (d) a satisfying state of being, and (e) feeling secure.

**Conclusions:**

This study demonstrates that a sense of home can be achieved in long-term care facilities for older adults with disabilities through the provision of systematic environmental and humanistic support. The findings reveal that a sense of home is collectively fostered through multiple dimensions, including quality caregiving, tailored physical environments, meaningful social interactions, and attention to individual needs.

**Supplementary Information:**

The online version contains supplementary material available at 10.1186/s12877-026-07379-w.

## Introduction

Since the 21st century, China has experienced a shift towards an aging society. According to the Seventh National Population Census, which is the country’s most authoritative demographic data source, the older population aged 65 and over has grown to 190.6 million [1], representing 13.5% of the total population. This growth rate has outpaced that of the younger children groups and the total population. With the increase in older adults comes a rise in the number of older adults with disabilities [2]. Older adults with disabilities refer to older adults who lose the ability to take care of themselves due to disease or aging [3]. Currently, China has approximately 46.5 million older adults aged 60 and above with disabilities, with a disability prevalence rate of 16.2% among this age group [4]. Demographic studies [5] predict that by 2030, the number of older adults with disabilities in China will exceed 77 million. With the aging of the population and the rising prevalence of age-related functional impairment, the need for daily care among older adults, especially those with chronic illnesses or limited mobility, has become an increasingly pressing social issue in China.

Currently, care services for older adults with disabilities are primarily provided through two models: home care and institutional care [6]. Traditional Confucian filial piety culture emphasizes the obligation of adult children to provide comprehensive care for their aging parents, encompassing physical assistance, financial support, and emotional comfort. Nevertheless, against the backdrop of social change and economic transformation, family structures have undergone a marked shift, characterised by shrinking household sizes and the predominance of nuclear families consisting of parents and unmarried children. These changes have gradually eroded the ethical function of home care, rendering the traditional filial piety model increasingly inadequate for meeting diverse modern care needs [7]. In response, the nationally implemented Long-Term Care Insurance (LTCI) policy serves as a critical institutional support. Funded jointly by the government, individuals, and society, LTCI provides subsidies for care costs, which not only alleviates the financial burden on families but also fosters the development of a professional care system through improved service quality [8]. Concurrently, China’s culture of care for older adults is undergoing a profound transition: the traditional notion of filial piety is evolving into a modern care culture that emphasises “social care complemented by filial support,” advocating shared responsibility between family and society [9]. Driven by these interconnected social, policy, and cultural factors, institutional care is gaining increasing acceptance among Chinese families. It is increasingly viewed as a viable approach to fulfilling filial duties, leading growing numbers of older adults with disabilities to spend their later years in long-term care institutions [10].

Older adults with disabilities spend most of their time in long-term care facilities due to limited mobility and reduced social activity. Such a facility is more than just a place that provides care; it is a long-lasting place for them to settle down [11, 12]. Therefore, it is essential to cultivate a warm and homely environment for them [13]. Creating a home-like atmosphere was also found to be particularly important to the quality of life of long-term care facility residents in Davis’s study [14], which is a common theme in healthcare policy worldwide [15].

In recent years, the focus of architectural design in care institutions has broadened from merely addressing functional needs to include addressing residents’ emotional experiences, integrating humanistic care [16] and configuring environments that cultivate a homely experience [17, 18]. A home, however, is not merely a functional space; it is a realm that safeguards privacy and self-expression and serves as a center for family reunion, belonging, and emotional connection. The sense of home, defined as the experience of belonging and comfort [19], is a multifactorial phenomenon. It is influenced not only by the built environment but is also profoundly shaped by social and personal characteristics and is inextricably linked to individual experiences and emotions. Consequently, fostering a sense of home must begin with older adults themselves, which emphasizes the importance of understanding their perspectives and ideas.

Yet, contemporary international research on the sense of home in care facilities primarily focuses on older adults who maintain a level of independence in their daily lives [20–22], paying limited attention to the experiences of those with disabilities. In parallel, studies within China on long-term care facilities generally emphasize the supply and demand for care services [23–25], while fewer delve into the lived experiences of older adults in these environments. This study specifically targets older adults with disabilities residing in Chinese long-term care facilities to explore their genuine experiences and sense of home. The goal is to provide valuable insights to healthcare professionals, enabling long-term care service providers and caregivers to determine the most appropriate services and care options, and to better implement the concept of person-centered care.

## Aim

To explore and describe the lived experience of a sense of home among older adults with disabilities residing in long-term care facilities, and to understand the factors that shape this experience.

## Methods

### Study design

Employing a descriptive phenomenological approach [26], this study conducted individual interviews to explore the lived experiences of older adults with disabilities in long-term care facilities. Guided by the principle of returning to the things themselves [27], this methodology requires researchers to bracket pre-existing assumptions and theoretical presuppositions. This process enables direct engagement with and faithful description of phenomena as they appear in consciousness, thereby revealing the essential structures of lived experience. This methodological approach is highly aligned with the study’s objective to explore and describe the lived experience of a sense of home among older adults with disabilities in long-term care institutions and to understand the factors shaping this experience. The study strictly adhered to the COREQ (Consolidated Criteria for Reporting Qualitative Research) guidelines [28] (Supplemental Material 1).

### Study setting

This study was conducted at a medium-sized private long-term care institution in East China that is an accredited provider under the Chinese Long-Term Care Insurance scheme. All long-term care institutions in China must comply with unified national standards governing operation and accreditation, particularly regarding staffing, environmental facilities, and spatial configuration [29, 30]. The selected institution strictly adheres to current national policies and development standards for the care of older adults while implementing a person-centered care philosophy [31], making it broadly representative of comparable facilities nationwide [32].

The institution occupies approximately 16,533 square meters with a capacity of 600 beds and maintains an average occupancy rate of 90%. It provides tiered care services based on older adults’ independence levels, housing autonomous and care-dependent older adults in separate residential buildings. The facility incorporates comprehensive barrier-free and age-friendly design principles, equipped with a 24-hour emergency call system, dedicated rehabilitation training areas, and multi-purpose common spaces. The caregivers-to-resident ratio is approximately 1:5, and registered nurses are on duty to ensure the professionalism and continuity of care services.

### Participants

Participant selection was conducted using purposive sampling, combining the principle of theoretical saturation [33] with maximum variation sampling. From May to July 2024, we recruited older adults with disabilities residing in the facility, seeking to include individuals with diverse socio-demographic characteristics. Inclusion criteria were: (1) age 60 years or older; (2) minimum stay duration of one year; (3) a disability level classified as mild or above (score ≥ 1) based on the Chinese Long-term Care Insurance assessment instrument; and (4) provision of informed consent for voluntary participation. Exclusion criteria were: a history of severe psychiatric disorders, or the presence of cognitive, language, or hearing impairments that would hinder effective communication. Disability levels were determined using the standardized assessment instrument established under China’s Long-term Care Insurance policy, which evaluates nine functional domains: mobility, self-care ability, sleep and mental status, emotional state, pain level, interpersonal communication, cognitive function, sensory ability, and social participation [34]. The classification defined: 0 as no disability; 1–40 as mild; 41–90 as moderate; 91–150 as moderately severe; and 151–200 as severe disability.

### Data collection

Data were collected through in-person interviews with eligible older adults. The interview guide was initially developed based on research questions and literature review, and then refined through pilot interviews with two older adults with disabilities and group discussions(Supplemental Material *2*). Data from pilot interviews were not included in the data analysis. During her internship at the long-term care institution in East China from March to July 2024, the first author established a trusting relationship with potential participants, identified those who met the eligibility criteria, and obtained informed consent from both interviewees and their families. Interviews were conducted one-on-one in a quiet space, such as an interview room. Before the formal interviews, the researcher explained the study’s background, purpose, and significance. All interviews were audio-recorded with participant consent and lasted approximately 20–30 min. Data collection continued until data saturation was achieved. After the first 16 interviews, preliminary analysis indicated that the thematic structure was stable. To rigorously confirm that no new themes would emerge and to ensure the robustness of our saturation point [35], we conducted two additional interviews as a confirmatory step. These interviews were fully transcribed, coded, and systematically compared against the established thematic framework. This comparative analysis confirmed that no new codes or themes emerged, thereby providing robust verification that data saturation had been achieved. We wish to emphasize that all participants’ contributions were integral to the study; specifically, the final two interviews played a vital role in validating the completeness and stability of our thematic framework, giving us methodological confidence to conclude data collection.

### Data analysis

Data analysis proceeded concurrently with data collection. To ensure transcription accuracy and facilitate the immersion process essential for phenomenological analysis, all interviews were transcribed within 24 h after completion. The first author transcribed the recordings verbatim and promptly annotated the transcripts based on the interview context, creating memos and writing reflective diaries. NVivo 12.0 supported the organization of codes and themes during data analysis. The textual data transcribed from each interview were imported into the NVivo12 project. Colaizzi’s seven-step method [36] was employed for inductive analysis due to its systematic framework and phenomenological appropriateness. This approach effectively guided theme extraction and meaning reconstruction from interview transcripts, thereby providing robust support for illuminating the essential nature of the phenomenon under investigation. The specific steps are shown in Table 1. Two researchers (first and second authors) independently conducted reading, coding, categorization, and theme extraction. In cases of disagreement regarding the themes, a third researcher (the third author) was invited to join discussions, ensuring that all parties reached a consensus to uphold the authenticity of the findings. To enhance the credibility of the data analyses, the researchers collaborated through group discussions to develop themes pertinent to this study and clearly defined the final thematic results.

**Table 1:** Colaizzi’s seven-step process for qualitative data analysis

NO	Data analysis step
1	The interview data were repeatedly and carefully reviewed to achieve thorough familiarity with the information provided by the participants.
2	A line-by-line analysis was conducted to identify, highlight, and extract meaningful statements directly related to older adults with disabilities’ perception of a sense of home in long-term care institutions.
3	The meanings of all significant statements were summarized.
4	Themes were clustered by grouping recurrent and meaningful ideas into coded categories reflecting common concepts.
5	A comprehensive description of the phenomenon of sense of home among older adults with disabilities in long-term care institutions was developed.
6	Similar subthemes were identified, essential structures were delineated, and main themes were derived.
7	The resulting thematic structure was returned to participants for feedback and validation to ensure credibility.

### Rigor

This study employed a phenomenological approach and ensured its rigor by adhering to established criteria for trustworthiness, including credibility, dependability, confirmability, transferability, and reflexivity [37].

1. Credibility: All interviews were audio-recorded in their entirety, and detailed notes were maintained. The researcher, who has received systematic training in qualitative methods, established a trusting relationship with participants prior to the interviews to facilitate the acquisition of authentic and in-depth information. Throughout the interviews, the researcher maintained a neutral stance and employed techniques such as repetition, probing, follow-up questions, and summarization to advance the dialogue. Care was taken to avoid premature interpretation or subjective assumptions, striving to faithfully represent participants’ original meanings.As a final validation step, we conducted member checking by returning the preliminary thematic structure to all participants for their feedback.
2. Dependability: The research clearly delineates the entire process of data collection and handling, including the specific procedures for note-taking and audio transcription, to ensure transparency and traceability of the research process.
3. Confirmability: Multiple strategies were used to enhance the objectivity and neutrality of the findings. In addition to continuously writing reflective memos, multiple researchers were involved in data analysis and coding. Cross-discussion and comparison were conducted to reduce biases arising from individual subjective judgments.
4. Transferability: The presentation of findings includes ample direct quotations from participants and detailed contextual information. The research context, participant characteristics, and data collection environment are thoroughly described to enable readers to evaluate the applicability and transferability of the conclusions to other settings.
5. Reflexivity: Prior to data collection, all authors engaged in introspection to document their assumptions, preconceptions, and personal values. This preliminary exercise enabled the researchers to consciously bracket their biases during data collection and analysis. Throughout the research process, the team held regular discussions and collective reflection sessions to ensure that the analysis remained grounded in participants’ actual experiences and narratives.

### Ethical considerations

This study was conducted in strict accordance with the ethical principles outlined in the Declaration of Helsinki and received ethical approval from the Ethics Committee of Xuzhou Medical University (XZHIUz-24106). Recognizing the vulnerability of the older adults with disabilities participating in this study, a dual informed consent process was adopted [38]. In addition to obtaining oral or written consent from each participant, written consent was also secured from their guardians or close family members. All data were anonymized to protect personal privacy strictly [39]. Throughout the interview process, the principles of respect for autonomy and avoidance of harm were rigorously upheld. Participants were informed that they could withdraw at any time without any negative consequences, thereby ensuring their well-being was prioritized.

## Results

The findings from the 16 interviews indicated that most older adults felt at home in the facility. Among the participants, twelve were female and four were male; their detailed demographic profiles are presented in Table 2. Notably, participants who had stayed in the facility for a longer duration often described a deepening sense of being at home. Overall, five main themes and twelve sub-themes were identified from the analysis, as detailed in Table 3.

**Table 2:** Demographic characteristics of the participants

Participants	Sex	Age	Education level	Occupation	Living years	Degree of disability
P1	M	72	High school	Civil servant	2	Mild
P2	M	72	Junior high school	Worker	4	Mild
P3	M	73	Primary school	Worker	2	Moderate
P4	F	79	Primary school	Farmer	1	Mild
P5	F	74	Primary school	Farmer	4	Moderately severe
P6	F	73	Primary school	Worker	4	Moderately severe
P7	F	76	Primary school	Worker	1	Mild
P8	F	73	High school	Worker	3	Mild
P9	F	65	High school	Civil servant	5	Mild
P10	F	82	Primary school	Worker	5	Severe
P11	M	69	Primary school	Worker	3	Moderately severe
P12	F	78	High school	Teacher	7	Moderate
P13	F	85	Primary school	Worker	1	Severe
P14	F	78	Junior high school	Civil servant	6	Mild
P15	F	81	Primary school	Worker	4	Moderate
P16	F	65	Primary school	Worker	3	Mild

**Table 3:** Themes and sub-themes

Themes	Sub-themes
1. Self-identity	a. Voluntary admission to long-term care facilities b. Self-acceptance: accepting your incapacities
2. Gaining a sense of belonging	a. Placement of personal belongings b. Love and care from family members
3. A warm atmosphere	a. Care and respect from caregivers b. Accompaniment and communication from residents living together
4. A satisfying state of being	a. Regular routine b. Eat and sleep well c. Fulfilling activities
5. Feeling secure	a. Fully equipped b. Professional nursing

### Theme 1: Self-identity

This study found that whether older adults with disabilities can establish a positive self-identity after moving into a long-term care institution serves as a prerequisite for experiencing a sense of home. According to Zhao et al. [40], such an identity entails that, even within a new environment, older adults are still able to view themselves as the active subjects of their lives, capable of embracing change and rediscovering meaning in living. Only when this positive self-identity is fostered can an unfamiliar dwelling begin to be imbued with the distinctive sense of belonging, security, and comfort that defines a home.

#### Voluntary admission to long-term care facilities

Voluntary relocation emerged as a key factor in fostering a sense of home. Some participants described this decision as a rational and proactive choice made under realistic circumstances, regarding themselves as active agents rather than passive recipients in the process. In their accounts, this sense of agency was closely associated with holding positive expectations about the institution and with the experience of becoming physically and emotionally settled. The following quotations illustrate how a voluntary relocation decision facilitated a positive institutional experience:

(*P14:My partner and I are getting older and struggle with our legs*,* feet*,* and backs*,* making it hard to cook for ourselves. We spend all our time at home without help*,* and living with our kids would add stress for everyone. After discussing it*,* we decided to find a nice place for older adults*,* and it’s been a good move that we’re glad we made.)*

*(P7: I asked to be admitted to the nursing home myself. I can’t eat together with my wife*,* we have different tastes and neither of us can cook. I thought I can’t go out and buy it every day*,* I’ll just go into the nursing home*,* and I won’t have to worry about my meals.)*

In contrast, some participants who were admitted involuntarily typically faced greater adaptive challenges. They largely attributed their placement to perceived abandonment by family or to constraints arising from a sudden health crisis. From their descriptions, a profound sense of powerlessness and resentment permeated their experience, making it difficult to view the facility as a place of trust or to ever feel at home within it. Their concept of home remained firmly tied to their past, sustaining a psychological stance where the new environment could not be embraced as home. This complex experience is vividly captured by P4’s account:

*(P4: There’s no way*,* I have to live here even if I don’t want to*,* it’s something that’s not of my own volition*,* compulsive*,* but I feel in my heart that it’s not a home in here*,* that I didn’t want to come and live in this institution on my own.*)

#### Self-acceptance: accepting your own incapacities

Participants’ accounts revealed that accepting bodily decline could constitute a deeper level of self-identity, often connected to maintaining dignity and meaning. They described how this process of acceptance and self-reconciliation was associated with moving beyond isolation and toward greater participation in the facility’s social life and activities. The experience described by P16 illustrates this phenomenon:

*(P16:My children are busy with their own lives and can’t care for me. I’ve accepted that I can’t walk anymore*,* and staying in a care home is best for everyone. I get the support I need and enjoy talking with other residents. I think it’s important to face reality. Now I joyfully use my wheelchair to participate in activities every day — it’s even easier than when I walked! )*.

### Theme 2: Gaining a sense of belonging

The acquisition of a sense of belonging is a crucial theme in the process through which older adults with disabilities transition from feeling like temporary residents to experiencing a sense of home within the unique environment of a long-term care institution. This sense of belonging does not refer merely to physical residence, but rather denotes the individual’s perception of themselves as meaningful members within the institutional setting, accepted emotionally and valued within a web of social relations.

#### Placement of personal belongings

Carrying personal belongings is essential for creating a sense of home, as evidenced by participants’ active efforts to personalize their living spaces with objects imbued with personal memories and emotional significance. For them, this was a way to counteract feelings of unfamiliarity and foster a sense of belonging. Participants described these items as extensions of their past lives and identities, a connection that supported a transition from seeing the space as an impersonal room to experiencing it as a warm, personal home where they could feel at ease. P9’s account illustrates this role of personal belongings in making the facility feel familiar:

*(P9:Then we stay here for a long time as our home*,* and we have all our stuff here*,* and it’s set up like a home*,* and every time we go back to our room after doing an activity*,* we all say we are going home.)*

When most of an older adult with disabilities’ personal belongings are in a long-term care facility, their original home can become unfamiliar and uncustomized. For some participants, this transition fostered a growing sense of belonging within the institution, which they began to regard as their true home. This shift is exemplified by participants who expressed a distinct reluctance to return to their former residences, a sentiment powerfully captured in P13’s account:

*(P13:Sometimes my kid picked me up for the day and let me crash at her place overnight. Honestly*,* I don’t even want to; I’m not used to it. All my stuff is here now*,* and it’s not even convenient being at their place. I’ve asked them to just send me back to the care facility to sleep.)*

#### Love and care from family members

Family care served as an important source of emotional support for participants. They reported that regular contact and demonstrated concern from relatives eased psychological burdens and sustained their feeling of being cherished. Their accounts often framed these positive interactions as weaving an emotional link between the facility and their family lives. Some participants specifically noted that when family members shared pleasant time with them within the facility (such as walking in the garden or chatting in their rooms), it contributed to their gradually regarding the place as a home and a natural part of life. In this way, as P10’s experience shows, the facility could become interwoven with a sense of home through family presence:

*(P10: When I first came here I was homesick*,* but when my daughters came to visit me every day and talked to me*,* I didn’t feel homesick anymore. I now feel that this is my home*,* there is no difference between being here and being at home*,* and my kids came by to see me a lot.)*

### Theme 3: A warm atmosphere

This theme is described by the participants as an overall atmosphere that feels “comfortable,” “free from loneliness,” and “lively.” The creation of such an atmosphere does not rely on expensive facilities, but is rooted in the frequent and warm daily interactions and emotional exchanges among members within the institution.

#### Care and respect from caregivers

Many participants expressed that the attentive care and concern shown by their caregivers made them feel at home, highlighting their relationship with caregivers as profoundly influencing their overall experience in the facility. In their descriptions, care that extended beyond routine duties, such as proactive attention to personal needs, was what most fostered a sense of home and evoked a feeling of being cared for by family. This is illustrated by P12:

*(P12: The nursing staff are very concerned about us. I like to sleep with my feet sticking out at night*,* so they will take a closer look at me when they check in at night*,* and when they see me with my feet outside*,* they will cover me with a quilt*,* which is very attentive*,* and it makes me feel cozy and warm inside.)*

Conversely, unpleasant interactions with caregivers tended to intensify their aversion to the institutional environment:

*(P1: The caregiver who looks after me during the day is so unpleasant — I can’t stand listening to him talk. It frustrates me every day. I really don’t feel that he shows any concern for us older residents. But since my children aren’t able to care for me*,* I have no choice but to stay here. How could anyone feel at home under such conditions? )*.

In the participants’ accounts, the respect shown by caregivers permeated all areas of care, encompassing both daily routines and leisure activities. They emphasized that having full decision-making autonomy was what allowed them to live according to their familiar and preferred ways. This experience of respect was inseparable from feelings of equality, freedom, and the preservation of their dignity. P7’s narrative illustrates this connection well:

*(P7:I feel that human beings and human dignity are fully represented here*,* they respect our choices and it feels free and unfettered to be here.)*

#### Accompaniment and communication from residents living together

Participants reflected that even before moving to the facility, they had often experienced loneliness while living with family, as physical limitations prevented them from going out and gradually distanced them from their social circles. After relocating, they described finding a community of peers who shared similar ages and life experiences. Within this community, they were able to offer each other a unique form of understanding and comfort, one that they felt was difficult for family members or caregivers to replicate. This mutual accompaniment and communication manifested in daily activities such as engaging in conversations in common areas, exchanging life stories, and sharing feelings. The following accounts illustrate these peer interactions and positive experiences described by participants:

*(P7: I feel that there are a lot of people here*,* and we all live here like a big family*,* hot and lively*,* and if there is anything to say*,* there is someone to say it and someone to listen to it. At home*,* I can’t go out and have no one to talk to*,* which makes me feel very lonely.)*

*(P11: I feel happier here than at home. There are people to talk and chat with*,* and when we don’t have activities*,* we just sit in the lobby in the afternoon to chat*,* tell jokes*,* and feel joyful.)*

### Theme 4: A satisfying state of being

Within this theme, satisfaction does not refer to material luxuries or external conditions, but rather to a psychological sense of stability, contentment, and fulfillment that older adults with disabilities experience in their daily lives.

#### Regular routine

When describing their sense of home, participants stressed the importance of a regular daily routine. They noted that the rhythm of life in the care facility felt familiar, resembling the routines they had followed at home. Maintaining this routine was associated with feeling more in control of their lives and experiencing greater mental stability. The sense of stability and fulfilment fostered by a regular routine is exemplified in the narrative of P16:

*(P16:My daily routine is pretty much the same*,* and since I’ve done this at home before*,* I’m adjusting really quickly. I’m just taking it day by day*,* and honestly*,* time feels like it’s flying by.)*

#### Eating and sleeping well

During the interviews, participants repeatedly emphasized that having enough to eat and rest were of paramount importance to them. They described how the certainty of enough food and rest alleviated bodily anxiety, creating a peace of mind that they associated with home. Furthermore, the familiar acts of eating and sleeping themselves sometimes evoked bodily memories of past family life. In their accounts, the simple acts of eating and sleeping well did more than meet basic needs; they became a direct source of this everyday ease and a tangible link to the familiar sensations of home. Together, this ease and this link were integrated into their experience of an environment where both body and mind could settle. The following accounts from participants illustrate this perspective:

*(P8:I think as long as I eat well and sleep well here*,* I’m happy*,* and after living here for a long time*,* I now consider this place as my home. )*.

*(P5:My husband has passed away*,* and there is no one at home to care for me. I don’t want to go to my son’s house and disturb their life. I prefer to stay here*,* where I can eat well and sleep well. I am satisfied with this situation and have no desire to go anywhere else; I just want to remain here.)*

Moreover, on the premise that the basic need of having enough to eat was met, some participants expressed the importance of autonomy and a sense of control over their dining experiences. Despite acknowledging the general quality of institutional meals, the*y* indicated that limited food choices impeded their sense of being at home, as P1 noted:

*(P1: The food tastes good*,* but it would be better if we could choose for ourselves. At home*,* I could eat whatever I wanted*,* but here we have to eat whatever the kitchen prepares.)*

#### Fulfilling activities

Participants described a significant change in their daily activities after moving to the facility. They reflected that while mobility problems had often limited their participation in community life at home, group activities became a central and valued part of their new routine. They recounted how caregivers would assist by taking them in wheelchairs to common areas for activities such as learning songs, doing handicrafts, or painting. Many highlighted that these daily group sessions were what they most looked forward to, valuing them for the enjoyment they experienced and the renewed interest in previous hobbies that these activities provided. P9 and P16 share their accounts of this fulfilling experience: (P9:*Some people ask me if I’m bored here*,* and honestly*,* I’m not at all! We read books*,* chat with friends and family*,* and the time just flies by. There are activities every day*,* and they’re all different*,* so we join in and have a blast together! )*

*(P16: You see*,* I’m not even very mobile*,* and I don’t have any needs. What I look forward to the most each day is hanging out with everyone for group activities. We get together to sing*,* draw*,* and sometimes do some crafts. This wall is packed with my own handicrafts*,* which I used to enjoy making.)*

### Theme 5: Feeling secure

For older adults with declining physical function and multiple health risks, their original homes often become unsafe due to the lack of adequate physical support and immediate medical response. As a result, their expectation for a new living environment is to obtain a sense of certainty and reliable shelter. This feeling of security is not a one-dimensional concept; rather, it is constructed through the assurance provided by the physical environment and reinforced by trust in professional support.

#### Fully equipped

Participants emphasized the importance of safety features such as non-slip flooring and handrails. They described how these installations alleviated their fundamental anxiety about falling, which fostered a sense of psychological and physical security that was often absent at home. Within this secure environment, their confidence in engaging with the surroundings grew, renewing their sense of control over daily life. This enhanced personal agency, they conveyed, was integral to developing a sense of belonging and experiencing the facility as home. As P6 notes, these installations provided the reassurance needed for independent activity:

*(P6:I can’t really do much at home since my legs don’t move around that easily. It’s way easier for me to be here because I don’t have to worry about slipping on the floor*,* and I can get to the bathroom on my own without needing help.)*

Building on the sense of safety, participants also pointed to the importance of an accessible environment. They described how the absence of architectural barriers, like thresholds, not only allowed them to move independently through doorways and common areas in their wheelchairs but also expanded their daily activities, promoted familiarity with their surroundings, and fostered a greater sense of control. P16’s account describes how this freedom shaped daily experience:

*(P16:I can get almost anywhere I want to go here*,* and unlike home*,* there are no thresholds here*,* so it’s easy for me to get in and out in my wheelchair.)*

#### Professional nursing

For participants, the constant availability of professional nursing care forged a profound sense of safety. The 24-hour presence of registered nurses was described as creating a protective atmosphere that they likened to the security of a family home, a reliable refuge where they felt consistently “watched over.” This assuredness of care, the knowledge that help was always at hand, fostered a deep psychological security. It was this experience of being securely cared for that they identified as a a key contributor to their sense of belonging to the facility. P14’s account captures how this reliable support shaped their experience of feeling at home:

*(P14:I really like that I feel safe here. There’s always someone around*,* so if I need help*,* I just have to shout and someone will come. At home*,* I don’t feel that way. The kids are out most of the day*,* and sometimes I fall and can’t get back up*,* just stuck lying there until they come home.)*

P13’s account illustrates that routine health monitoring also constituted a significant source of psychological security within the long-term care setting:

*(P13:I feel pretty relaxed living here*,* and the kids are pretty chill too. Whenever I’m not feeling great*,* I just ring the bell*,* and a nurse shows up to check on me. I also often check my blood pressure and blood sugar during my free time.)*

## Discussion

This qualitative study aimed to explore and describe the lived experience of a sense of home among older adults with disabilities in long-term care facilities through an investigation of its presence and influencing factors. The findings revealed that establishing a self-identity is the initial step in perceiving a care facility as a home. Additionally, the placement of personal belongings and the presence of family can foster a sense of belonging in institutional settings. Companionship from caregivers and fellow residents creates a warm atmosphere, while having a satisfying living situation and a sense of security will also help older adults with disabilities feel at home.

Self-identification serves as a crucial indicator of an older adult’s acceptance and adaptation to a long-term care institution [41]. According to Erikson’s theoretical framework, self-identity refers to the internal psychological construct through which individuals maintain a sense of personal continuity while navigating significant life transitions, accepting reality changes, and rediscovering meaning in life [42]. For older adults with disabilities living in the institution, this process represents an active psychological adaptation, at its core involving the transformation of an external relocation decision into an internal sense of autonomy, while integrating the reality of physical disability into a still meaningful life narrative. The establishment of positive self-identity helps older adults regain life confidence and approach institutional care with an open mindset. It is noted in the literature [43] that a significant factor in helping an older person feel at home is their involvement in the initial decision to move into a long-term care facility, particularly whether they entered the facility voluntarily [44]. Participants who voluntarily chose institutional living described their experience as involving the reframing of their residence as a positive decision based on practical considerations, a process that supported the maintenance of self-continuity throughout role transitions. This phenomenon reflects the ongoing modernization of China’s older adult care culture [45, 46]. Within the context of evolving social perspectives and supportive mechanisms like Long-term Care Insurance, the self-identity rooted in such autonomous decision-making provides a crucial psychological foundation for experiencing the unfamiliar care setting as a place that could become a home.

However, traditional Chinese culture places significant emphasis on the belief that " Older adults should be properly cared for until the end of their lives, and when they pass away, they should be sent off with due respect” [47]. Influenced by the traditional concept of “raising children for old-age support” [45], many older adults with disabilities hold deeply ingrained attitudes regarding home care. They often resist long-term care institutions, perceiving institutional residence as indicative of family discord or lack of filial piety from their children, and view these institutions as places for the homeless or those abandoned by their families. Consequently, although some older adults with disabilities may be compelled to enter care institutions for physical or familial reasons, they commonly maintain a negative outlook on life within these facilities.

Adjustment to a new home is a process that occurs over time, with individuals responding differently, and there is no set time when familiarity begins and ends. For older adults with disabilities who choose institutional living voluntarily, the experience often involves pre-existing expectations regarding their new environment [48]. Furthermore, the favorable initial impressions resulting from their autonomous decision facilitate a more positive adjustment to and acceptance of life within the care institutions. Therefore, we suggest that long-term care institutions should step up publicity and education to increase older adults’ understanding of their policies and services, change their stereotypical impression of care institutions, and improve their resistance. Additionally, it is crucial to fully respect the wishes and choices of older adults when they decide whether or not to live in the institutions and provide older adults with support for their gradual transition to long-term care institutions, such as short-term residential experiences so that they can gradually adapt to the institutional environment.

Physical decline and environmental shifts render older adults with disabilities especially susceptible to self-identity crises and a diminished sense of self-worth [49]. They frequently struggle to accept the reality of physical limitations and experience role uncertainty, often developing a perception of themselves as “burdens to others” after institutionalization. These internal conflicts disrupt the continuity of their lived experience and challenge the establishment of a stable sense of belonging within the institutional setting [50]. Meanwhile, this study observed that institutionalized older adults with disabilities’ life expectations demonstrate a distinct shift toward focusing on basic survival needs. Rather than pursuing the spiritual or material achievements they might have valued in healthier times, they increasingly concentrate on fulfilling fundamental physiological requirements. During the interviews, some participants mentioned that they felt satisfied as long as they could have food and drink in the institutions. Further analysis found that for these physically vulnerable older adults with disabilities, adequate nutrition and stable sleep serve purposes beyond mere physiological maintenance: they function as crucial means to achieving physical security and alleviating existential anxiety. The fulfillment of these basic needs provides them with the psychological energy and emotional stability necessary for positive mental adjustment [51].

Within the context of functional decline as a significant life transition, positive mental adjustment serves as a crucial pathway for older adults in navigating encountered challenges and restoring meaning to their lives [52]. The group activities within long-term care institutions provide a significant platform for facilitating mental adjustment and play an essential role in promoting both psychological and physical well-being [53]. Gerontological theory [54] indicat*e*s that regular activity for older adults is the basis and key to maintaining self-esteem, psychological fulfillment, and a long and healthy life. Participation in activities can stimulate positive emotions, impart meaning to the lives of older adults, and evoke a sense of home [55]. By actively engaging in facility life, older adults with disabilities gradually develop emotional attachment and belonging to their environment, transforming from passively awaiting time’s passage to having anticipated daily pursuits [56]. Many participants in the study expressed that participating in activities was their most anticipated daily event. Previous studies [22] have reached similar conclusions: the continued participation of older people in care settings will help to give meaning to their reshaped daily lives.

Group activities also offer a valuable platform for older adults with disabilities to connect and share with one another, fostering opportunities for bonding with their peers. The commonality of similar ages and physical conditions among older adults with disabilities in long-term care facilities often creates a space of shared understanding. Within group activities, this shared ground makes the exchange of personal stories and emotional narratives a meaningful occurrence, which in turn fosters a palpable sense of emotional resonance and facilitates the formation of friendship among them. Establishing new social connections within institutional setting contributes to an improved sense of well-being and mitigates feelings of isolation [57], while also strengthening their sense of home [58]. Such interactions help older adults with disabilities feel valued and needed, as well as fostering a sense of belonging to an extended institutional family where they experience understanding and acceptance.

In addition to the companionship of peers, the care from family members and the support from caregivers are equally vital. The findings of this study indicate that the sense of home experienced by older adults with disabilities in institutions is closely intertwined with supportive relationships among family, peers, and caregivers. This observation aligns with prior research [59]. As defined by the American Geriatrics Society [60], person-centered care (PCC) is achieved through dynamic relationships among older adults themselves, individuals who are significant to them (such as family members), and all care providers (such as caregivers). Similarly, Koren’s theory emphasizes [61] that supportive social relationships between residents, family members, and nursing staff are necessary for person-centered care, which is recognized internationally as a key principle in long-term care [62]. Molony et al. found [63] that in nursing home settings, closeness to and involvement with family are associated with a sense of being at home. Interactions with family serve as an important source of emotional support for older adults with disabilities. When they maintain close ties with their families, they can more easily accept and adapt to living in a care facility [43]. Strong relationships with family members help people feel connected to the outside world, support continuity between their past and present, enhance their sense of belonging within the long-term care facility, and improve the quality of life for older adults with disabilities [64].

In the daily life of care facilities, the positive interactions with caregivers also serve as an important source of emotional support. Participants in this study viewed their relationships with caregivers as the most significant factor influencing their overall long-term care experience. This finding is corroborated by Cooney’s research, which also identified the resident-staff relationship as central [44]. For older adults with disabilities, who often contend with feelings of helplessness and inferiority due to declining abilities, being understood and valued by caregivers emerged as particularly crucial. Consistent with the literature on dignity in care [65], the respect demonstrated by caregivers was experienced by participants as reinforcing their sense of worth, thereby boosting self-esteem and self-confidence [65]. Furthermore, through daily care and communication, caregivers provide emotional companionship and support that alleviates loneliness and fosters a sense of warmth akin to home.

The interviews revealed that for participants, personalizing their living space with objects imbued with personal memories and emotional significance was a way to counteract feelings of unfamiliarity and foster a sense of belonging. This finding not only corroborates previous research highlighting the importance of personal belongings in cultivating and sustaining a sense of home [66] but also aligns with Blackler’s perspective that being surrounded by items imbued with meaningful memories enhances environmental familiarity and facilitates adjustment to new surroundings [67]. Notably, participants described these items as extensions of their past lives and identities, a connection that supported a transition from seeing the space as an impersonal room to experiencing it as a warm, personal home. As noted in Lewinson’s study [68], the ability to bring personal history into space through beloved objects and cherished furniture may contribute to a sense of home.

For a care facility to become a home, it must provide an environment where residents feel safe, secure, and comfortable. The interviews highlighted that some participants perceived the facility as offering greater security than their private homes, largely due to accessible features such as elevators, handrails, and threshold-free design. They described how these well-equipped environments supported independent mobility for wheelchair users, enabling them to actively navigate their surroundings. This active engagement with the physical space facilitates a transition from viewing the facility as an impersonal setting to experiencing it as a familiar territory. Literature suggests that when older adults have the opportunity to explore their new environment and become familiar with its every corner, they begin to feel that it truly belongs to them [22]. Long-term care facilities in China are also required to have a level of medical care that ensures continuous health monitoring and timely intervention in case of disease recurrence, thereby better safeguarding the health of older adults with disabilities. This provision of responsive professional care complements environmental security by addressing the core physical vulnerability concerns of older adults with disabilities, thus constituting the critical link in transforming a physically safe space into an emotionally felt home. 

Moss, Davies, and Brown-Wilson [69] question the appropriateness of characterizing long-term care facilities as “homes,” noting residents’ limited autonomy. This study’s findings align with this concern, as some participants reported that the inability to choose their meals hindered their sense of being at home. They expressed a desire for daily food selection rather than accepting institutional decisions. Although acknowledging satisfactory meal quality, many believed that dietary autonomy would contribute significantly to feeling at home and maintaining control over their daily lives. This finding is consistent with the recognized importance of independence to the lived experience of nursing home residents [70]. Restricted food options can undermine self-determination and autonomy, potentially compromising quality of life [71]. Additionally, disregarding residents’ food preferences may lead to adverse health outcomes including unintended weight loss and malnutrition. Therefore, we recommend that care facilities implement practical measures to personalize meals. Specifically, this can be achieved through initiatives such as establishing next-day meal pre-ordering systems, providing standardized yet adaptable menus tailored for common health conditions (e.g., hypertension and diabetes), and maintaining individual dietary preference profiles. These practical measures aim to empower residents with meaningful choice and support adherence to their health needs, thereby enhancing autonomy and overall well-being.

### Limitations

Firstly, the use of a single-site sampling strategy may limit the diversity of the sample, as all participants were recruited from one facility located in East China. Although this sample has a certain degree of representativeness, the findings are inevitably shaped by the specific cultural, regional, and managerial context of this facility. Therefore, the transferability of these findings relies on the reader’s judgment of contextual similarities. The results may offer insights for interpreting similar phenomena in comparable settings, but their applicability to environments with distinctly different operational mechanisms, resource levels, or regional cultures would require careful consideration. Secondly, the purposive sampling approach may have led to the overrepresentation of older adults with disabilities who were verbally expressive, potentially underrepresenting the perspectives of those with cognitive or communication impairments. Furthermore, the average interview duration of 20–30 min, though intended to reduce physical and emotional burden on frail older participants, may have limited the depth of experiential exploration. To mitigate this, interviews were highly focused and employed open-ended probing techniques. Thematic analysis indicated that core themes reached saturation despite the time constraints. Finally, as with any qualitative study, subjective interpretation and recall bias are inherent challenges. It should also be noted that due to the historical reasons of the era, most participants had relatively low levels of formal education, which may have affected their ability to fully comprehend interview questions and articulate their views, thereby potentially influencing the depth and quality of the data.

## Conclusion

The sense of home is inherently subjective. While previous studies have suggested that long-term care institutions can never truly replace one’s home, this study reveals that, through systematic environmental and humanistic construction, most older adults with disabilities can still develop a sense of home within institutional settings. This research indicates that the sense of home is not determined by a single factor, but rather arises from the interplay of multiple dimensions, including caregiving approaches, environmental features, social interactions, and individual needs. Caregivers should demonstrate respect and care, and actively promote social interaction to help older adults build a supportive environment and meaningful relationships. At the same time, service providers should strive to gain a deep understanding of older adults’ needs and fully respect their daily habits and autonomy in decision-making, thereby establishing a physical and psychosocial environment that is truly home-like. Furthermore, the ongoing involvement of family members, along with proactive adaptation on the part of the older adults themselves, remains an essential component in fostering a sense of home. This study offers valuable insights into the perceptions of older adults with disabilities about the sense of home in a long-term care facility, with important implications for improving their quality of life in long-term care.

## Supplementary Information

Supplementary Material 1. COREQ checklist.

Supplementary Material 2. Interview guideline.

## Data Availability

The data generated and analyzed during the current study are available from the corresponding author upon reasonable request.
