# Supplementary material for: Do long-term care facilities feel like home? Views from older adults with disabilities
Source: BMC Geriatr. 2026 Mar 25;26:626. doi: 10.1186/s12877-026-07379-w (PMC13137563; doi:10.1186/s12877-026-07379-w)
Supplement: Supplementary file 2 — Supplementary Material 2. Interview guideline. [file 12877_2026_7379_MOESM2_ESM.docx]

Interview guideline

1. Can you tell us why you chose to live in a long-term care facility at that time?

2. Can you tell us about your daily life in the facility?

3. How is your diet, personal hygiene and sleep here? Do the facilities and services here meet your needs?

4. How do you usually spend your time here? Do you participate in social or recreational activities?

5. Do you feel at home in the care home? Can you be more specific about what makes you feel that way?

6. What is ‘home’ to you and what does it mean to you? What elements or feelings do you think are most essential to a home?

7. How is it different from your previous home? How does this affect you?

8. Are you satisfied with your current living situation? Can you tell us why?

9. Do you feel that your privacy and dignity are respected here?

10. Do you feel cared for and supported by the carers here?

11. How often does your family visit you? Do you miss them here?

12. If you have a health problem at the facility, do you receive timely and effective treatment and care? Are you satisfied with the treatment and care?

13. Do you feel that these places (respect, care, etc. mentioned above) make you feel at home?

14. Do you have any suggestions for measures or service improvements for the organisation where you live, so that the elderly can feel more at home and their quality of life can be improved?
